# Effects of High-Intensity Progressive Resistance Training Combined With Self-Talk on Muscle Strength and Functional Performance in Older Adults

**DOI:** 10.1519/JSC.0000000000005115

**Published:** 2025-04-02

**Authors:** Vittoria Ferrando, Francesco Mirabelli, Marco Panascì, Denise Sofrà, Piero Ruggeri, Emanuela Faelli, Ambra Bisio

**Affiliations:** 1 Department of Experimental Medicine, Section of Human Physiology, University of Genoa, Genoa, Italy;; 2 Centro Polifunzionale di Scienze Motorie, University of Genoa, Genoa, Italy;; 3 Department of Neuroscience, Rehabilitation, Ophthalmology, Genetics, Maternal and Child Health, University of Genoa, Genoa, Italy

**Keywords:** incremental load, motivation, elderly, mobility capacity, strategy

## Abstract

Ferrando, V, Mirabelli, F, Panascì, M, Sofrà, D, Ruggeri, P, Faelli, E, and Bisio, A. Effects of high-intensity progressive resistance training combined with self-talk on muscle strength and functional performance in older adults. *J Strength Cond Res* 39(7): 736–744, 2025—In older people, muscle mass and functional capacity decline, affecting balance, and gait control. In this population, resistance training (RT) improves muscle strength, counteracting this physiological decline. In younger individuals, cognitive strategies like motivational self-talk have been successfully integrated with RT to enhance its effectiveness. This study aimed to compare the effects of a high-intensity progressive RT with positive motivational self-talk against RT alone on muscle strength and functional performance in older adults. Twenty-eight healthy older people were divided into resistance training self-talk (RT-ST) group and RT group. The RT-ST group mentally repeated positive motivational phrases during lower-limb exercises, whereas the other group performed RT alone. The 4-week protocol included biweekly sessions, consisting of warm-up, a high-intensity resistance exercise, and cooldown. Handgrip strength, 1 repetition maximum (1RM), 30-second chair stand (30 s-CS), and time up and go (TUG) tests were conducted before (PRE), immediately after (POST), and 2 weeks after follow-up (FU) of the intervention. One repetition maximum significantly increased from PRE to POST (*p* ≤ 0.001) and FU (*p* ≤ 0.05) in both groups. The 30 s-CS significantly improved only in the RT-ST group at FU (*p* ≤ 0.001), with higher values compared with the RT group at POST (*p* ≤ 0.05) and FU (*p* ≤ 0.05). Time up and go test duration decreased in both groups (*p* ≤ 0.001), with RT-ST completing the test faster than RT (*p* ≤ 0.05). Incorporating positive motivational self-talk into a high-intensity progressive RT program led to significant improvements in functional performance, suggesting that its benefits go beyond improving muscle strength and may positively impact the activities of daily living in older adults.

## Introduction

Age-related changes in the human body lead to various dysfunctions, including altered mobility and physical disability, resulting in a reduced sense of independence and overall quality of life (3). In particular, the decline in sensorimotor function and alterations in the nervous system contribute to a decrease in muscle strength, power, balance, and functional performance (3). A previous study reported that 23% of people between ages 60 and 69 years reported 1 or more physical limitations that increased with age (8), with a reduction in type II muscle fibers, correlating with a rapid loss of muscle strength (36). Notably, the loss of muscle strength has been shown to be more closely related to the impairment of muscle power than muscle mass (17,28). These changes can affect rapid postural reactions in response to external perturbations, leading to a loss of balance and increasing the risk of falls up to 60% (17). In turn, falls or the fear of falling can reduce motor engagement and negatively impact activity of daily living (ADL), with implications for health, functional autonomy, and overall quality of life (15,28).

Resistance training (RT) has emerged as a promising strategy to counteract age-related functional decline (15). A recent review reported improvements in mobility, muscle strength, quality of life, and functional independence in older adults following RT interventions (15,28). One of the most studied forms of RT, even in older people, is progressive RT, which is characterized by working against loads that gradually increase in intensity. In older adults, progressive RT executed 2 or 3 times per week has been shown to reduce muscle weakness and some functional limitations (e.g., changes in balance and walking speed) (29). Liu and Latham (29) investigated the effect of different progressive RT protocols on lower-limb strength in an older population and the results suggested that a high-intensity training load promoted a greater increase in lower-limb strength than a low-intensity training load (29). However, caution is warranted, as adverse events such as pain during or after the exercise have been documented (7).

In young adults, the combination of a conventional RT with cognitive interventions has been demonstrated to increase training effectiveness, mainly physical function, strength, and performance (4). One of these interventions is self-talk. Self-talk is a technique used during mental skills training to regulate cognitions, emotions, attitudes, and performance (45). Self-talk is a cognitive manipulation defined as “an internal dialogue [in which] the individual interprets feelings and perceptions, regulates and modifies evaluations and convictions, and provides instructions and reinforcement to self” (16). Self-talk serves as a tool to increase motivation and focus and is commonly used to improve athletic performance. Although numerous studies have investigated the effect of self-talk on performance in sports (20), little attention has been paid to assessing its potential effectiveness in improving balance and other basic ADLs. Depending on its content, self-talk can be either motivational or instructional (10). Hardy et al. (19) found that motivational self-talk is more effective for tasks that require strength and endurance, whereas instructional self-talk is more effective when the task requires precision and timing. Furthermore, the valence of self-talk can be positive or negative. The former refers to a self-talk that is positive in content (e.g., praise and encouragement), which builds self-confidence, creates positive moods, helps people to concentrate, and increases motivation (21). Negative self-talk proposes negative content, such as criticism and self-absorption (10), which can sometimes mislead the person who is using it and thus create blocks (46).

Based on the results obtained in young adults, it can be hypothesized that positive motivational self-talk applied during a conventional RT in older adults may increase its effectiveness in improving strength and functional performance, but no data are available. This easy-to-use tool could improve the efficacy of RT in the elderly, thus helping them to increase the benefits they receive. Because completing a resistance exercise can be particularly challenging for older adults, likely because of a variety of barriers (i.e., safety concerns, fear, health problems, pain, fatigue, and lack of social support) (15), it is possible that the addition of this cognitive technique could increase their confidence and motivation to exercise and help them feel better and stronger. With the aim of identifying a new strategy to enhance the efficacy of conventional RT in the older population, this study investigated the effects of a high-intensity progressive RT program combined with a positive motivational self-talk on lower-limb strength and functional performance in active older people.

## Methods

### Experimental Approach to the Problem

The study was a 4-week, randomized, controlled trial with an experimental group (resistance training self-talk; RT-ST) and a control group (RT). The duration of the intervention period was chosen based on the results of Del Vecchio et al. (11) who showed that 4 weeks of strength training in adults was sufficient to induce significant neural adaptations underlying the increase in muscle strength. In addition, our elderly subjects worked at very high intensities (90% 1 repetition maximum [1RM]), and some of them also underwent simultaneous cognitive training. For these reasons, the training period was limited to 4 weeks to avoid the risk of overtraining, overexertion, and, consequently, dropout. The study began with a subject interview in which the health status was investigated, the training protocol was explained, and the informed consent form was completed and signed. During the explanation, all subjects received information provided from the researcher about the exercises they would be performing, and only the RT-ST subjects were instructed to use positive motivational self-talk by mentally repeating a phrase they found motivating while executing the exercises.

**Figure 1.**
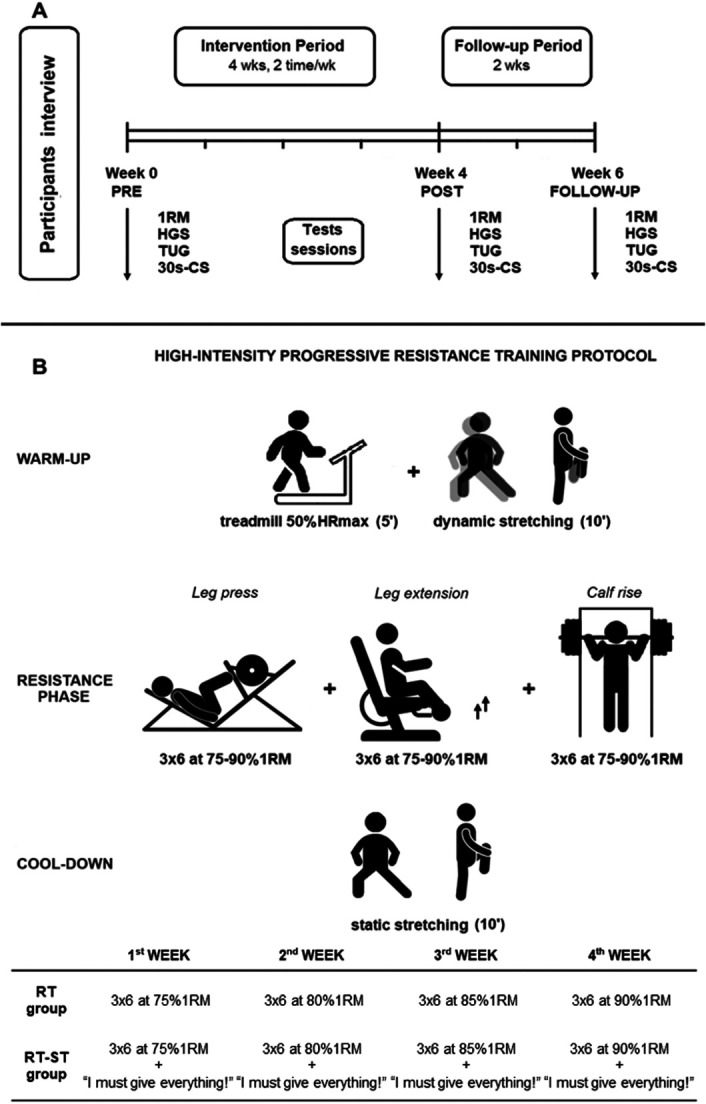
A) Subjects underwent a preliminary interview in which they received explanation about the training and signed the informed consent. Before (PRE), after (POST) intervention period and after 2 weeks (follow-up, FU), subjects underwent to 1 repetition maximum (1RM), handgrip strength (HGS), time up and go (TUG), and 30-seconds chair stand (30 s-CS) tests. B) Both resistance training self-talk (RT-ST) and resistance training (RT) intervention period lasted 4 weeks (2 times/week); each session consisted of: warm-up, resistance phase during which subjects performed exercises on leg press, leg extension and multipower machines, and cool-down. The table at the bottom of the figure shows the training load in each group.

During the intervention period, both groups performed 8 training sessions, twice per week with a total duration of approximately 60 minutes. Before (PRE), at the end of the intervention period (POST), and 2 weeks after the end (follow-up, FU), subjects underwent strength and functional performance assessments. Resistance exercises and tests were performed using machines to facilitate movement control during the exercise. All training sessions and tests were one-to-one sessions carried out between a graduate sports science researcher and the subject in the Sports Science Laboratory at the University of Genoa, Italy. Figure 1A provides a graphical representation of the experimental protocol.

### Subjects

An a priori estimation of sample size was made based on data from the 30-second Chair Stand (30 s-CS), which represents a reliable and valid indicator of the functional performance and lower-body strength in active older adults (26,40). Sample size was estimated using the GPower software (3.1 software Düsseldorf, Germany), applying repeated measures ANOVA (*F* Test) with a significance threshold of *α* = 0.05, a statistical power of 90% to an effect size (ES) of 0.7, and a margin for possible refusals or dropouts of 35%. This calculation generated a minimum sample size of 18 subjects in total (13).

The eligibility criteria were (a) age older than 60 years; (b) absence of artificial limb and neurological and cardiovascular disorders, orthostatic hypotension, musculoskeletal diseases, or injuries, prosthesis within 6 months before the beginning of the study; (c) absence of hip or ankle arthrosis and any other contraindications for physical activity; (d) during the intervention and FU periods, subjects were prohibited from performing any other training outside of the current training; and (e) being active according to the World Health Organization (older adults who are engaged in at least 150 minutes of moderate-intensity physical activity per week) (9). Subject characteristics at the baseline are presented in Table 1.

**Table 1:** Subject characteristics.*

Subjects (*n*°)	Age (yrs)	Body mass (kg)	Height (cm)	Body mass index (kg·m^−2^)	Muscle mass (kg)	Fat mass (%)
28	68.59 ± 5.05	67.04 ± 10.55	167.18 ± 8.5	23.9 ± 2.43	46.86 ± 8.85	26.5 ± 7.67

* The characteristics of the subjects at baseline are reported as mean ± *SD*.

Twenty-eight active older adults (mean ± *SD*: age 68.59 ± 5.05 years, from 61 to 80 years; 13 men and 15 women), not engaged in strength sport disciplines, were enrolled, and randomly divided into 2 groups: RT-ST (*n* = 14) and RT (*n* = 14) groups. In the RT-ST group, a positive motivational self-talk was administered throughout the RT exercise, whereas the RT group performed the RT exercise alone.

Subjects were instructed to arrive in a well-rested and adequately hydrated state, at least 3 hours after consuming a standardized meal, and to refrain from engaging in strenuous exercise within the 24 hours preceding each test session. In addition, they were asked to abstain from consuming caffeine and alcohol for 24 hours before the test. All training session were performed on leg press, leg extension, and multipower machines, in a laboratory with controlled temperature and humidity (21–24 °C and 44–56%, respectively).

The experimental protocol was conformed to the code of Ethics of the World Medical Association (Declaration of Helsinki), and it was approved by the Ethics Committee of University of Genoa (protocol code: 2023/14 and date of approval: February 16, 2023).

### Procedures

#### High-Intensity, Progressive, Resistance Training Protocol

Each training session lasted approximately 60 minutes, beginning with a 15-minute standardized warm-up, followed by an RT phase and ending with a 10-minute cooldown. The warm-up consisted of 5-minute treadmill running, followed by 10-minute dynamic stretching exercises focusing on lower-limb muscle groups (12). Treadmill intensity during the warm-up was set to maintain the individual at 50% of the maximum heart rate (13), predicted indirectly using according to the Karvonen’s formula (27). Heart rate was continuously monitored throughout the running exercise with a Garmin watch (FORERUNNER 15, Olathe, KS). Dynamic stretching exercises were chosen because they have been shown to be effective in older people for reducing passive muscle tension and increasing joint flexibility and blood circulation in the lower-limb muscles (47). The RT phase consisted of 3 sets of 6 repetitions of high-intensity progressive RT at 75–90% 1RM (see Strength Assessments section) of leg press, leg extension, and calf raise exercises, with 2 minutes of rest between the sets (18,43), which was sufficient to tolerate the protocol without symptoms. Specifically, the lifted load progressively increased by 5% 1RM each week, from 75% 1RM in the first week to 90% 1RM in the fourth week, maintaining 6 repetitions per set for each exercise. At the end of each session, subjects performed a 10-minute cooldown, including static stretching exercises focused on the lower-limb muscle groups most involved in the previous exercise (Figure 1B).

#### Positive Motivational Self-Talk Intervention

Before the start of the intervention period, the experimenter provided subjects in the RT-ST group with all the information about the effects of positive motivational self-talk published in the scientific literature. Subjects in this group were required to use self-talk throughout each RT exercise. They were asked to focus on a sentence of their choice that was the most encouraging for them, repeat it to themselves in an inner dialogue, and believe in its meaning. At the beginning, in the presence of the experimenter, they were given time to choose the best sentence for them, and they experienced the sentence while doing the proposed exercises to be convinced of its efficacy. The researcher emphasized to the subjects the importance of maintaining a high level of concentration during self-administration, as this could influence their awareness of task performance (10). At the beginning of each session, subjects were reminded to use self-talk, and at the end of each session, the experimenter asked them to confirm that they had used it during practice. All subjects in the RT-ST group always confirmed that they used self-talk during all the training sessions. Examples of sentence used by the subjects were “I am a Caterpillar machine,” “I have to train, here and now,” and “I have to give it my all.” During the postintervention assessments, POST and FU, the RT-ST group was instructed not to use the positive motivational self-talk to avoid its effects.

#### Strength Assessments

Handgrip strength (HGS) was chosen as a general measure of body strength in relation to the current and future health status, including specific outcomes of general strength, fractures, bone mineral density, falls, and mortality (6). Strength was measured using a Jamar hydraulic handheld dynamometer (Sammons Preston, Rolyan, Bolingbrook, IL), which allowed for standardized and objective evaluation of HGS, ensuring consistency of assessment technique across individuals. Subjects sat on a chair with their feet flat on the floor, holding the handgrip with their wrist in line with their elbows, and they were instructed to press the dynamometer as hard as possible. Subjects performed a maximal voluntary isometric contraction of the finger flexor muscles. To obtain accurate and reliable measurements, 3 separate trials were conducted with 10-second intervals between each trial. Both the dominant and nondominant sides of the body were assessed, and the mean value of the 2 body sides was calculated as the whole-body overall HGS test. The maximum values were considered for statistical analysis (36).

Maximum lower-limb strength was assessed by 1RM, which determines the individual's maximum lifted load using the same exercises employed during the training program: leg press, leg extension, and calf raise at multipower machines (Technogym Spa, Gambettola, Italy) (28,32). These 3 exercises were chosen as a measure of strength because they involve the main muscle groups of the lower body. The loads and repetitions were entered into the O'Connor equations, and an individual 1RM was determined (39). Each test was separated by 5 minutes of rest. Before the start of testing, subjects performed a 5-minute treadmill warm-up and dynamic stretching exercise (28).

#### Functional Performance Assessments

The 30-s CS was used to assess the functional performance because it is a measure of lower-limb muscle strength and relates to the most demanding activities of daily life (26,33). During the 30 s-CS, subjects sat in a standard chair (seat height of 40 cm), with their backs upright, their feet on a flat surface positioned about shoulder width apart, their arms crossed, and their hips and knees flexed at approximately 90°. At the researcher's command, they stood upright and returned to the starting position for as many repetitions as possible within 30 seconds. Subjects were encouraged to repeat as many of the sit-to-stand actions as possible, and the number of sit-to-stand actions completed was recorded (33).

To assess functional mobility, including dynamic balance and walking speed, subjects also completed the time up and go test (TUG). During this test, subjects were seated in a standard chair, and upon verbal cue from the researcher had to stand, walk 3 meters at their self-selected habitual walking speed, turn around a cone, and return to sitting (17).

#### Body Composition Assessments

Body composition was evaluated using bioelectrical impedance analysis (BIA; Tanita, BC-420 MA, Tanita, Tokyo, Japan) with a 50-kHz frequency measurement. The evaluation was performed on subjects who had fasted for 2 hours before arrival. Subjects were asked to stand barefoot with their arms at their sides and their legs and thighs not touching. The outcome parameters used to measure the body composition were body mass (kg), body mass index (BMI), fat mass (%), and muscle mass (kg).

### Statistical Analysis

The outcome parameters were HGS, 1RM leg press, 1RM leg extension, 1RM calf raise, TUG, 30-s CS, body mass, BMI, fat mass, and muscle mass. These data were checked for normal distribution by means of the Shapiro-Wilk test and for sphericity violation by Mauchly's test. Handgrip strength, 1RM leg press, 1RM leg extension, TUG, and body composition data (body mass, BMI, fat mass, and muscle mass) were normally distributed, whereas 30 s-CS and 1RM calf raise were not. Normally distributed data were statistically evaluated by means of ANOVA with TIME (3 levels: PRE, POST, FU) as within subject factor, and GROUP (2 levels: RT-ST, RT) as between-subject factors. Significant effects and interaction were assessed by means of Bonferroni’s post hoc tests. For non-normal distribution, comparison within group was done by means of Friedman’s tests, followed by post hoc, whereas comparison between the groups was performed by means of the Mann-Whitney test. Normally distributed data are provided as the mean ± *SD*, whereas non-normally distributed data are presented as the median associated with interquartile range. The significance level was set at *p* < 0.05. Partial eta-square value was used to quantify the ES (pƞ^2^). The statistical analysis was performed with SPSS (SPSS, Inc., Chicago, IL).

## Results

### Strength Assessments

Numerical results for the outcome parameters related to strength assessments are reported in Table 2. The statistical analysis on HGS revealed no significant effects of TIME and GROUP and no significant interaction. The results of 1RM leg press and 1RM leg extension revealed a significant effect of the factor TIME (1RM leg press: F(2,52) = 29.924, *p* = 0.0004, pƞ^2^ = 0.535; 1RM leg extension: F(2,52) = 43.703, *p* = 0.0002, pƞ^2^ = 0.627). Post hoc tests indicated that 1RM leg press values significantly increased from PRE to POST (*p* = 0.0003) and to FU (*p* = 0.0002). One repetition maximum leg extension significantly increased from PRE to POST (*p* = 0.0004) and to FU (*p* = 0.0003), and in POST, it was significantly lower than in FU (*p* = 0.021). No significant differences appeared between the groups in both parameters. The result of the Friedman’s test on 1RM calf raise showed a significant effect of TIME in both groups (RT-ST: χ^2^(2) = 26.14, *p* = 0.0005; RT: χ^2^(2) = 18.58, *p* = 0.0007), indicating a significant increase of strength from PRE to POST (RT-ST: *p* = 0.014; RT: *p* = 0.001) and from PRE to FU (RT-ST: *p* = 0.0008; RT: *p* = 0.0009). No differences were found between the groups.

**Table 2:** Strength, body composition, and functional performance assessments.*†

Variables	RT-ST group			RT group		
	PRE	POST	FU	PRE	POST	FU
Body composition						
Body mass (kg)	66.7 ± 7.7	66.6 ± 8.1	66.4 ± 8.1	65 ± 12.6	68.1 ± 14.7	67.4 ± 8.2
BMI (kg·m^−2^)	23.9 ± 1.8	23.9 ± 1.9	23.8 ± 1.9	23.5 ± 3.1	23.6 ± 3.1	23.3 ± 3.3
Lean mass (kg)	46 ± 7.9	4.2 ± 8.1	46.1 ± 8	45.6 ± 8.9	50.2 ± 11.37	39.7 ± 3.3
Fat mass (%)	27.8 ± 7.9	22.3 ± 7.1	28.3 ± 6.8	25.7 ± 8	22.3 ± 7.11	28.3 ± 6.8
Strength parameters						
HGS (kg)	26.44 ± 6.40	25.48 ± 6.03	26.76 ± 5.96	27.61 ± 10.37	26.78 ± 10.18	27.19 ± 9.91
1RM leg press (kg)	171.57 ± 30.32	220.62 ± 48.85	234.86 ± 45.88	162.86 ± 44.41	207.21 ± 57.82	197.55 ± 46.37
1RM leg extension (kg)	48.21 ± 11.98	62.75 ± 17.27	66.25 ± 16.66	44.24 ± 10.71	52.17 ± 14.89	55.07 ± 16.99
1RM leg calf (kg)	40.5 (32.5–54)	65 (57.5–101.75)	75 (59.93–104.06)	43 (31–57)	54.5 (43–81.25)	52 (43.75–81.5)
Functional performance evaluations						
30 s-CS	14.5 (11.98–18)	17 (16.08–18.25)	18.95 (17.75–20)	12 (11–15.28)	15.7 (11–17.25)	14.85 (12.75–17.5)
TUG (s)	7.72 ± 1.56	6.63 ± 1.09	6.17 ± 0.94	8.43 ± 1.37	7.4 ± 1.32	7.54 ± 10.48

* Subjects' body composition, strength, and functional performance parameters before (PRE), at the end of the intervention (POST) and 2 weeks after (follow-up; FU). Data a provided as mean ± *SD* in case of normal distribution, and median [interquartile interval], in case of not normal distribution.

† BMI = body mass index; HGS = handgrip strength; TUG = time up and go; 1RM = 1 repetition maximum; 30 s-CS: 30-s chair stand.

### Functional Performance Assessments

**Figure 2.**
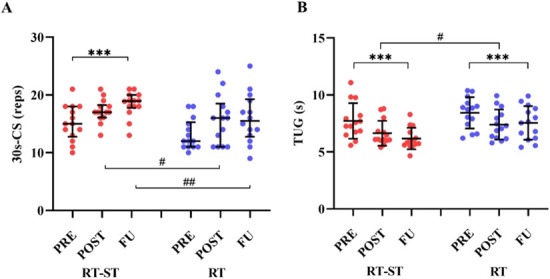
Results of the 30-second chair stand (30 s-CS) (A) and time up and go (TUG) (B) tests. Red and blue circles refer to single-data values of subjects in the RT-ST and RT group, respectively. In (A) the horizontal line indicates the median values, and the bars represent the inter-quartile ranges in 30 s-CS, whereas in (B) the horizontal line indicates mean values, and the bars show the standard error. *Indicates difference within group; ***p < 0.001. Differences between the groups are indicated by the ^#^symbol; ^#^*p* < 0.05; ^##^*p* < 0.01.

Numerical results for the outcome parameters related to functional performance assessments are reported in Table 2. The results of Friedman’s test on 30 s-CS values in RT-ST group showed a significant effect of TIME (χ^2^(2) = 14.38, *p* = 0.001). Post hoc revealed a significantly higher values in FU than in PRE (*p* = 0.001). No changes were observed in RT group. The Mann-Whitney test showed no difference between the groups in PRE, whereas significantly higher values were found in RT-ST compared with RT group in POST (*p* = 0.044) and FU (*p* = 0.014). Results are shown in Figure 2A.

The result of ANOVA on the time to complete the TUG test showed a significant effect of the factor TIME (F(2,52) = 25.809, *p* = 0.0005, pƞ^2^ = 0.498). Bonferroni’s post hoc revealed that the values significantly decreased from PRE to POST (*p* = 0.0003) and from PRE to FU (*p* = 0.0004). Furthermore, the time to complete the TUG test was significantly lower in RT-ST than in RT group (GROUP: F(1,26) = 4.464, *p* = 0.044, pƞ^2^ = 0.147). A graphical representation is offered in Figure 2B.

### Body Composition Assessments

Subjects' body mass, BMI, and lean mass were comparable between the 2 groups at baseline and did not change after the intervention period. Fat mass values showed a significant effect of the factor TIME (F(2,52) = 8.482, *p* = 0.001, pƞ^2^ = 0.246). Bonferroni’s post hoc test revealed a significant decrease in fat mass from PRE to POST (*p* = 0.042) and from PRE to FU (*p* = 0.001) in both groups. No differences appeared between the groups. Data are reported in Table 2.

## Discussion

This study investigated the effects of 4 weeks of progressive RT plus self-talk on strength, functional performance, and body composition in active older people. Specifically, the effects of a lower-limb, high-intensity, progressive RT combined with a positive motivational self-talk (RT-ST) were compared with those of the same RT alone. The results showed that self-talk improved functional performance as demonstrated through 30-s CS and TUG. In particular, in the RT-ST group, the number of repetitions of the 30 s-CS test increased significantly from the baseline, and values at POST and FU were significantly higher compared with those at the same time in the RT group. Regarding functional mobility, the time to complete the TUG test was significantly lower in the RT-ST group compared with the RT group. Furthermore, TUG time decreased significantly from the PRE to POST in both groups, and this effect was maintained at FU. Similar effects were seen in both groups concerning the maximal lower-limb strength on 3 different machines, which increased significantly after the intervention period. Finally, both the RT-ST and RT groups showed a significant reduction in fat mass after the 4 weeks, and this beneficial effect was further enhanced in the following 2 weeks after training, as shown by the FU assessment. Overall, in addition to the evidence reported above, this study was successful in terms of adherence and motivation among older adults, as indicated by no reported dropouts.

The importance of increasing the lower-limb strength in older people cannot be underestimated. Not only does it contribute to better postural control and balance but also it correlates with improved functional performance and independence in activities of daily living (14). In such a context, progressive RT was suggested by Plotkin et al. (37) as effective in improving both muscle endurance performance and maximal strength, which are essential for optimizing muscular adaptations. Other studies emphasized the importance of interventions targeting lower-limb strength to reduce fragility in older individuals, improve endurance, and reduce the risk of falls, thereby promoting independence (3,35). Furthermore, improvements in strength correlate with enhanced performance in daily activities, such as walking and climbing stairs (42), likely because of the improved voluntary muscle activation, which in turn may play a role in ameliorating the functional performance and mobility (30,31). Notably, a decline in calf muscle strength has been associated with reduced walking speed and balance, suggesting the critical role of lower-limb strength in maintaining mobility in older adults (1). This evidence may explain the current significant improvements in TUG and 30 s-CS. It is noteworthy that these improvements were achieved after a training period of only 4 weeks. Based on the study of Del Vecchio et al. (11), it could be speculated that these results are motivated by an increase in motor neuron drive to the muscle.

The results of the TUG test, a widely used measure of functional mobility, showed a notable decrease in completion time from PRE to POST assessments, reaching significance 2 weeks after the end of training (FU). It should also be noted that TUG values were significantly lower in the RT-ST group compared with the RT group, suggesting the additional benefit of the self-talk intervention to functional mobility.

Analysis of the 30 s-CS test proved to be a compelling indicator of the positive effects of self-talk applied to progressive RT in the older population. Only the RT-ST group showed a significant increase in the number of repetitions within 30 seconds. In addition, this group exhibited a significantly higher number of repetitions immediately after the intervention and at the FU assessment with respect to the RT group. These findings strongly suggest that the positive motivational self-talk enhanced the effectiveness of progressive RT, resulting in improved functional performance, particularly in tasks such as rapid standing and sitting within a given time frame. This result agrees with those of a previous study showing the positive effect of this cognitive manipulation on a time trial test. Indeed, an increase in distance travelled was found after positive self-talk during an endurance performance (5). It could be hypothesized that the inner dialogue enacted by the subjects through self-talk improves the attentional focus and attentional performance, thus preventing the loss of concentration (20), which are crucial elements for successful performance in functional performance tests and, more generally, in daily life activities (22). It should also be noted that the 30 s-CS test may represent a sort of competition with oneself for the older people, in which individuals try to outperform their own baseline performance. Hatzigeorgiadis et al. (23) proposed that the usefulness of motivational self-talk may rely also on increasing motivation and effort, especially in competitive contexts. Thus, the competitive nature of the 30 s-CS test may have been particularly sensitive to the effects of self-talk on motivation. In response to this suggestion, the positive motivational phrases that subjects mentally repeated were carefully selected to match the task and individual needs, likely optimizing the effectiveness of self-talk intervention.

One may also consider that self-talk acts on self-regulation, which has multiple dimensions, including self-reinforcement and self-management, which have been shown to correlate with each other (38). Therefore, the use of positive motivational self-talk may also have indirectly helped subjects to regulate the course of the action during training (for instance, on how to distribute energy expenditure), leading to more efficient action control. This speculation is supported by the neuroimaging studies showing that self-talk is associated with the activation of the ventromedial prefrontal cortex (34), which is an area involved in both self-control and decision making.

This result might also be attributed to the known abilities of positive motivational self-talk bolster self-confidence (22). The meta-analysis by Hatzigeorgiadis et al. (22) describes the correlation between increased self-confidence and improved task performance. Based on this report, it could be hypothesized that the increased self-confidence serves as a plausible mechanism underlying the beneficial effects of self-talk on task performance in the older population. This finding is consistent with previous studies in sports reporting the positive impacts of self-talk on performance, motivation, and feeling better and stronger (44). In addition, a final point to discuss is that, although the improvement in 30 s-CS performance was evident immediately after the end of training, it reached statistical significance in the FU period, suggesting the enduring impact of the self-talk intervention and its potential long-term benefits. These findings are also consistent with previous studies, showing that the effects of RT on muscle strength and functional mobility are maintained even after periods of detraining (2). One explanation for this improvement during the detraining phase could be that there was a large variability in how older people recover (24). Specifically, after the last week of loading at 90% 1RM, not all subjects may have fully recovered from the previous week's unloading. However, this should not have been the case, given the magnitude of the treatment effect (24,41), as the RT-ST group obtained improved POST and FU values and significant PRE and FU values in the 30 s-CS and TUG tests. Nevertheless, this aspect needs further investigation into future studies.

The results showed a significant reduction in the percentage of fat mass in older people after progressive RT, which was maintained at the FU assessment in both groups. Consistent with our findings, several studies have shown that RT can lead to significant improvements in body composition in older people, including reductions in body fat percentage (13,18,25). This supports the idea that strength training should be considered an important component of fitness and health programs for older people. These concepts were incorporated into our study, which used a personalized, progressive approach to strength training and achieved positive results in terms of improved fat mass percentage in older subjects. Furthermore, subjects in both the RT-ST and RT groups, who had no experience of RT, showed significant improvement in maximum strength (1RM) in the 3 exercises (leg press, leg extension, and calf raise) after the intervention period, and these values were maintained up to 2 weeks after (FU). These strength adaptations were achieved despite the low number of training sessions, suggesting effectiveness of the protocol. These findings are in line with the research of Plotkin et al. (37), who showed that the use of machines for RT resulted in optimal development of maximal muscle strength in both leg extensors and ankle plantar flexors.

It should be noted that these observed strength gains are consistent with those reported by Steib and co-authors using intensities up to 90% 1RM, although they are lower than those reported in previous studies involving low-intensity training protocols (<75% 1RM), thus supporting the effectiveness of the high-intensity load range in promoting significant neuromuscular adaptations (18,43).

Therefore, because both groups increased the 1RM, the self-talk intervention did not induce any surplus in terms of muscular adaptation with respect to those induced by the high-intensity progressive RT. One could speculate that the intensity was high enough to induce a very strong muscular adaptation that statistically masked the additive effect of self-talk on these parameters. The numerical results may give some indication of this aspect. In fact, although no significant differences were found between the groups, the increase in 1RM from PRE to POST in the RT-ST group was in the range of 29–59%, whereas it was lower in the RT group, in the range of 18–28%. It is likely that the effect of self-talk would have been more pronounced if lower training intensities had been used. Further studies are needed to confirm this hypothesis.

It should be noted that this study had limitations. First, we did not examine the effects of self-talk on perceived exertion, which some studies have suggested may influence performance (22,23). Second, even if 4 weeks was sufficient to see improvements, extending the training to 6–8 weeks, with a consequent redistribution of workload over more sessions during the intervention period, could probably produce more pronounced results. Finally, it would also be interesting to investigate the responses to different types of self-talk and whether different training loads would produce better adaptations.

This study represents the first attempt to investigate the impact of a self-talk intervention on the efficacy of progressive RT in older adults. Although the prescribed progressive RT alone resulted in significant gains in maximum strength for both groups, the addition of positive motivational self-talk yielded sustained benefits in functional performance that have a major impact on maintaining the individual's autonomy in ADL. Therefore, these findings suggest that high-intensity progressive RT should be an integral part of fitness programs for older people.

**Practical Applications**

This study showed that incorporating self-talk into a 4-week RT program promotes and consolidates the functional capacity adaptations in older adults. Specifically, this protocol could be used to improve the dynamic balance during walking and the lower-limb muscle fibers recruitment during activities of daily living, purportedly reducing the number of falls. Given the effectiveness and the ease of administration of self-talk, its use in adapted physical activity for healthy older adults should be promoted and incorporated by trainers into exercise regimens tailored for older individuals.
